# THE EFFECT OF DISPOSITIONAL OPTIMISM ON SUCCESSFUL AGING OF OLDER ADULTS AND THEIR PARTNERS

**DOI:** 10.1093/geroni/igz038.2648

**Published:** 2019-11-08

**Authors:** Gina Lee, Kyuho Lee

**Affiliations:** 1 Iowa State University, Ames, Iowa, United States; 2 Daegu University, Gyeongsan, Korea, Republic of

## Abstract

The purpose of the study was to examine how dispositional optimism of an older adult influences both the individual’s and the spouse’s successful aging. Data from the Health and Retirement Study were included in this analysis. The age of participants ranged from 18 to 104 (M = 67.91, SD = 11.26). The successful aging components included low levels of depressive symptoms and low levels of difficulties in activities of daily living, subjective health, and cognition. A structural equation model was computed including optimism as a latent variable and four components of successful aging for older adults and their spouses, all from the same wave in 2014. The results of the study revealed that higher dispositional optimism of older adults significantly predicted lower depressive symptoms, lower difficulties with activities of daily living, better cognitive function, and higher subjective health, for older adults and their spouses. The results, in conclusion, support the notion that dispositional optimism not only plays a significant role in well-being for oneself but also benefits the partner’s well-being as well. Further research need to include other components of successful aging, such as social engagement, loneliness, and life satisfaction. In addition, including covariates, such as gender, educational attainment, race/ethnicity, and household income, will also further examine the effect of optimism above and beyond the demographic factors.

